# Unrealized potential of drug repositioning in Europe during COVID-19 and beyond: a physician's perspective

**DOI:** 10.1186/s40545-020-00249-9

**Published:** 2020-07-17

**Authors:** A. B. Bayoumy, N. K. H. de Boer, A. R. Ansari, F. Crouwel, C. J. J. Mulder

**Affiliations:** 1 Faculty of Medicine, Amsterdam UMC, University of Amsterdam, Amsterdam, The Netherlands; 2 Department of Gastroenterology and Hepatology, Amsterdam UMC, VU University Medical Center, AG&M Research Institute, Amsterdam, The Netherlands; 3 Department of Gastroenterology, East Surrey Hospital, Surrey, UK

**Keywords:** Market Authorization Application (MAA), Centralized procedure (CP), Decentralized procedure (DCP), Mutual recognition procedure (MRP), Reference Member State (RMS), Drug repositioning, Drug rediscovery, Orphan drugs, Pharmaceutical compounding, Magistral compounding, Thioguanine, Thalidomide, Mexiletine, Chenodeoxycholic acid, Remdesivir, Hydroxychloroquine, COVID-19, SARS-COV 2

## Abstract

Drug repositioning is the scientific strategy of investigating existing drugs for additional clinical indications. The advantages of drug repositioning are that it benefits patients and that it adds new indications to existing drugs for lower costs compared to de novo drug development. Clinical research groups recognizing efficacy of these “old” drugs for a new indications often face an uphill struggle due to a lack of funding and support because of poor structural and regulatory support for clinical drug development. The current framework for drug repositioning allows “venture capital” companies to abuse loopholes in the legislation to gain long-term market authorization among with excessive high pricing. A new regulatory framework is needed to prevent abuse of the legislation and promote clinical investigator-driven drug repositioning. The COVID-19 pandemic has boosted funding and regulatory support for drug repositioning. The lessons learned from the COVID-19 pandemic should be implemented in a new clear blueprint for drug repositioning. This blueprint should guide clinicians through legislation for drug repositioning in the EU. This review summarizes the routes for registration and discusses the current state of drug repositioning in Europe.

## Introduction

Drug discovery marked important events in the history of medicine. The discovery of anesthesia made it possible to perform complicated operations, and the discovery of penicillin by Fleming has led to a breakthrough in modern medicine [1]. In spite of the common interest of the whole society, today, drug development is almost exclusively pharma led. The reason for this includes prohibitory costs on the journey from drug development, data authorities need for registration, and results of preclinical tests and clinical trials. The resulting near monopolization of this entire process by pharma means that it is almost impossible to find the regulatory expertise to comply with the legal requirements for marketing authorization. This classical drug development is now such a costly enterprise which is difficult for noncommercial organizations such as university research institutes or academic hospitals to perform.

Unlike drug development, *drug repositioning* is the scientific strategy of investigating existing drugs for new therapeutic purposes [2]. Because the drugs’ toxicity and pharmacokinetic properties are already understood the route to authorization via drug repositioning can be less costly than de novo drug development. On top of the advantage of cost savings, the clinical safety profile of the drug can be based on “real-world” clinical data and on the post-marketing pharmacovigilance reporting. The costs for authorization may therefore be reduced significantly and may provide clinicians a wide-array of new treatment modalities for their patients. However, successful drug repositioning also depends on additional factors including clinical resemblance of the disease (e.g., anti-inflammatory drug for auto-immune disease), patient demographics, and other features of therapy (e.g., dosage and combination with other drugs). If these conditions are different, it can increase the costs of drug repositioning because of the need of additional data.

Clinical research groups recognizing the efficacy of these old and often off patent generic drugs need funding and regulatory support for patient benefit through the process of clinical drug development. However, pharmaceutical firms are not obliged and often not inclined to facilitate or support non-commercial efforts for drug repositioning. Therefore, unlocking the potential of drug repositioning is lost due to lack of support for these clinical groups. Our review summarizes the routes for registration and discusses the current state of drug repositioning in Europe.

## Different procedures for registration in Europe

First, it is important to understand how the procedures for drug registration work in Europe. The main legal basis for drug applications for drug repositioning in the European Union (EU) can be found in the Directive 2001/83/EC. Market access of medicinal products (any substance or combination of substances presented as having properties for treating or preventing disease in human beings) in the EU is regulated through legislation by setting standards for safety, quality, and efficacy.

The main objective of the pharmaceutical legislation in the EU is to protect uncontrolled movement of medicinal products and to safeguard public health. A marketing authorization is required for all medicinal products before entering the EU market. During the marketing authorization procedure, the regulatory authority assesses the data provided by the applicant, in which the product satisfies these criteria of quality, safety, and efficacy.

The application process for drug repositioning in the EU is based on three different procedures for submitting a Marketing Authorization Application (MAA) in the EU:

1. The centralized procedure (CP)
2. The decentralized procedure (DCP)
3. The mutual recognition procedure (MRP)

The summary of the three application procedures can be found in Table 1.

**Table 1:** Summary of the three application procedures in the European Union

	Central procedure	Decentral procedure	Mutual recognition procedure
Authorization authority	European commission	National	National
Registration location	All 27 EU member states	One or more EU member state(s)	Multiple EU member states
Application	Simultaneous in all states	Separately in each individual member state	Simultaneous at one or more concerned member states
Existing approval	No existing approval in EU member state	No existing approval in EU member state	Existing approval by EU member state national market authority (Reference Member State)
Compulsory	Yes, for innovative medicines (incl. orphan disease)	No	No
Data exclusivity and market protection	8 years data exclusivity + 2 years market protection (+ 1 year market protection for new indication)		

### Centralized procedure

The European Medicines Agency (EMA) is responsible for execution of the centralized procedure, which allows to put a product on the market in all 27 EU member states and in Norway, Iceland, and Liechtenstein. Submission to one MAA thus leads to one assessment process and one authorization that allows access of the market of the entire EEA (European Economic Area).

The centralized procedure is mandatory for certain products as mentioned in the Regulation 726/2004:

1. High technology medicinal products, particularly those resulting from biotechnological processes.
2. Orphan medicinal products, these are products registered for orphan diseases. An orphan disease is defined as any disease affecting fewer than 5 people in 10,000. Furthermore, the centralized procedure is also mandatory for any medicinal product for human use containing an entirely new active substance, i.e., one that has not yet been authorized in the EU.

### Decentralized procedure

In the decentralized procedure, applicants can apply for simultaneous authorization of a drug in more than one EU member state, if it has not yet been authorized in any EU country and does not fall within the scope of the centralized procedure. The applicant has to find an authority that has an open slot for assessment. The Reference Member State (RMS) that assesses the submitted MAA will provide the other selected member states with the conclusions and results of the assessment. If positive, then the drug can be authorized in all the selected EU member states.

### Mutual-recognition procedure

If an applicant company already has applied for, or obtained market authorization in another EU member state, the national and decentralized procedures are not available. For this instance, the mutual-recognition procedure is required. The RMS can apply for this authorization to be recognized in a chosen Concerned Member States (CMS). This process allows member states to rely on each other’s scientific assessments and could lead to authorization in the CMS. However, this route cannot be used for drug repositioning because the drug has not yet been authorized for the new indication.

## Different pathways for drug repositioning in Europe

Applicants for a new active substance need to submit the latest safety and efficacy data, while companies, organizations, or individuals producing known active substance are allowed to submit “dossiers” that contain previous data. This can be done without the need for a new clinical trial by referring to previous or other “dossiers.” The two important pathways for drug repositioning are the complete dossier pathway and the well-established use pathway.

### Complete dossier pathway

The complete dossier pathway, which can be found in Article 8(3) of Directive 2001/83/EC, can be considered the standard application pathway. It is composed of general administrative information, complete (non)-clinical data based on the applicants’ tests and studies, and/or bibliographic literature substituting/supporting certain tests or studies.

### Well-established use pathway

The well-established use application pathway, which is provided in Article 10a of Directive 2001/83/EC, can be used for drugs that have been used for at least 10 years in the EU, with recognized efficacy and an acceptable safety level. The applicant is not required to provide the (non)-clinical data in the dossier but may use existing literature. In addition, applicant’s own data may be used to prove similarity of the applicant’s product to the product used in the literature.

### Generic/hybrid application pathway

A generic medicine has the same active ingredients as the authorized reference medicine; it may only be produced after expiry of the data exclusivity period. When a manufacturer aims to develop a generic drug but it differs in dosage strength, indication, or pharmaceutical form, then it must follow the hybrid application pathway. The authorization of these hybrid drugs may refer to the results of the authorized reference medicine but may also provide new data from clinical studies. The legal basis of the hybrid application can be found in Article 10(3) of Directive 2001/83/EC.

## The financial incentives for drug repositioning in Europe

The European legislation provides the holders of a marketing authorization with regulatory protection in the form of data exclusivity and market protection. Data exclusivity is the period of time during which a generic (or hybrid or biosimilar) application cannot cross-refer to the data in support of marketing authorization for the reference medicinal product (i.e., generic, hybrid, and biosimilar). Such applications will be refused by regulatory authorities during period of data exclusivity. Market protection refers to the period of time during which generic, hybrid, or biosimilar cannot be placed on the market, even if the medicinal product has already received marketing authorization before.

The European Medicines Agency (EMA) follows the 8 + 2 (+ 1) exclusivity formula, in which the first 8 years are data exclusivity, followed by 2 years of market protection. There are also provisions available that could extend market protection and data exclusivity:

- + 1 year market protection for a new therapeutic indication which brings significant benefit in comparison with existing therapies (Art.14(11), Reg. (EC) No 726/2004)
- + 1 year data exclusivity for a new therapeutic indication for a well-established substance, provided that significant pre-clinical or clinical studies were carried out in relation to the new indication (Art. 10(5), Dir.2001/83/EC)
- + 1 year data exclusivity for a change in classification of a medicinal product on the basis of significant pre-clinical test or clinical trials (Art.74(a), Dir. 2001/83/EC)

**Fig. 1:**
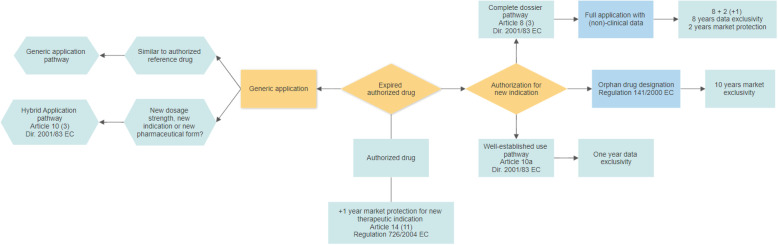
Algorithm for drug repositioning pathways in the European Union

The algorithm for drug repositioning pathways can be found in Fig. 1.

## Successful examples of drug repositioning

Although history has many successful examples of drug repositioning, there is the important cautionary example of thalidomide, which as an anti-emetic in pregnancy, caused congenital malformations. The thalidomide disaster resulted in approximately 2000 deaths and serious birth defects in over 10,000 children leading to its withdrawal in 1961 [3]. As a consequence, stricter regulations were initiated, and all new drugs had to be properly “authorized” before being placed on the market. In 1964, thalidomide was prescribed as sedative in a patient who also had erythema nodosum leprosum (ENL). In this patient, the ENL skin eruption disappeared within 48 h [4] and led to positive clinical studies for this new indication. Thalidomide was reintroduced for this complication of leprosy and supported by the World Health Organization [5]. By 1989, there were additional benefits of thalidomide discovered for several HIV complications such as oral ulceration, wasting syndrome, and Kaposi’s sarcoma [6]. Due to the continued interests in thalidomide, the drug remained available for controlled clinical trials and compassionate use treatments. In 1998, the Food and Drug Administration (FDA) approved thalidomide for the treatment of ENL, which contributed to the availability of the drug for other diseases [7]. In 1994, anti-angiogenic properties were first described using a rabbit cornea model of FGF-induced neovascularization [8]. These anti-angiogenic properties were later confirmed in other studies [9, 10]. The discovery of the anti-angiogenic properties has boosted interested in thalidomide as an anti-cancer therapy. Plasma cell myeloma is associated with high serum VEGF concentrations and increased bone marrow microvascular density [11]. Several clinical studies were performed to assess the clinical effectiveness of thalidomide in plasma cell myeloma [12, 13]. Thalidomide, in combination with dexamethasone, was approved as orphan drug treatment for plasma cell myeloma in May 2006. Thalidomide can be seen to have found multiple useful indications in the past six decades and was designated as orphan drug then approved through the centralized procedure [14].

A more recent example is thioguanine, which was originally developed and licensed for the treatment of leukemia in the 1950s [15]. In the following decades, it has been investigated for a wide-range of inflammatory diseases such as psoriasis and systemic lupus erythematosus [16]. In 2001, thioguanine was found to be a promising rescue therapy for patients with inflammatory bowel disease (IBD) who failed mercaptopurine or azathioprine treatment [17]. In the Netherlands, thioguanine has been conditionally registered as rescue therapy for IBD after failure of conventional thiopurines since 2016. However, authorizing this drug was complicated mainly because a blueprint for authorizing an off-patent drug for a new indication was lacking. There was also little interest from the pharmaceutical industry to invest in authorizing thioguanine due to an unattractive cost-recovery (only 1 year of market exclusivity when licensed). Thioguanine was approved through the well-established use pathway in the Netherlands [18].

## Off-label prescription as consequence of missing incentives for drug repositioning

In clinical practice, there are many examples of off-label drug use [19], especially in the field of oncology or for special populations (e.g., pediatric patients and geriatric patients) [20]. Off-label use can be defined as any intentional use of an authorized product not covered by the terms of its marketing authorization and therewith not in accordance with the SmPC. Whereas market approval of medicinal products is the subject to EU legislation and falls under the responsibility of the national competent authorities or, in case of a centralized procedure, the European Commission (EC), EU legislation does not regulate the use of medicinal products in daily clinical practice. Off-label prescription can offer important advantages for the individual patients: it provides new treatment options, especially when approved treatment options have failed. Furthermore, it also gives patients and clinicians earlier access to drugs and the adoption of new practices based on newly obtained evidence. However, a major disadvantage of off-label uses the generally low level of evidence regarding safety and efficacy. It was found that of all off-label drugs only approximately 20% is supported by strong evidence [21]. Other limitations of off-label use are the increased responsibility [20, 22] (e.g., legal claims, risk-benefit analysis of evidence, and implicit informed consent) put on the prescribers and pharmacists, issues with reimbursement (i.e., special application or no reimbursement), as well as undermining of the drug authorization system of prior approval before marketing. There is a need for labelling these off-label drugs for the sake of patient safety. As these drugs are not formally licensed for the indication, there is no safety surveillance for the drug on that indication.

Lack of reimbursement for off-label drugs for new treatment indications vary from country to country in Europe. Often, off-label use of drugs is not reimbursed. However in daily clinical practice, especially for cancer and HIV drugs, in contrast to auto-immune diseases, reimbursement of these off-label drugs is possible due to strong media and political support.

In major markets such as Europe and the USA, a clinician should preferably prescribe a drug by reference to the generic name. However, due to strong pharmaceutical marketing, physicians usually refer to brand names. The prescription will be filled by a pharmacist without knowledge about its medical indication. The drug will then be reimbursed at a fixed level regardless of whether a branded or generic version is dispensed. Thus, most reimbursement systems nearly always provide a financial incentive to the pharmacist to dispense, when available, a (cheaper) generic drug. This is also called the preference policy, which is mandatory in some major markets such as Germany [23]. For pharmaceutical companies, there are so far limited attractive financial incentives for drug repositioning. Legislation only allows for 1 year extra market protection if a new indication is added in the first 8 years after a marketing authorization has been granted, and the new indication must have significant clinical benefit over existing therapies. However, off-label prescription will continue to occur without good incentives for the drug repositioning of “older” drugs, especially in off-patent products for which drug repositioning will not likely result in return for on investment.

## Orphan disease: high prices and exploitation of regulation

In recent years, drug development has shifted from blockbuster to “niche buster” due to improved incentives for orphan drugs [24]. The regulation particularly designed for these orphan drugs is the Orphan Regulation 141/2000EC. To receive an orphan drug designation, the drug must meet three criteria:

1. The drug must be intended for the treatment, prevention, or diagnosis of a disease that is life-threatening or chronically debilitating;
2. The prevalence of the condition in the EU must not be more than 5 in 10,000, or it must be unlikely that marketing of the drug would generate sufficient returns to justify the investment needed for its development;
3. No satisfactory method of treatment, prevention, or diagnosis of the condition concerned can be authorized, or, if such a method exists, the drug must possess a significant benefit to those affected by the condition.

The orphan regulation declares that a drug that has been labeled as an orphan drug by the Committee for Medicinal Products for Human Use receives market exclusivity for a period of 10 years. Also, there is an opportunity for a 2-year extension if a pediatric investigation plan is included. One medicinal product may have more than one orphan disease designation. The orphan drug designation can be allocated to products approved through either the 8.3, 10.3, or 10(a) pathway.

All orphan drugs must follow the centralized procedure (EC regulation No 726/2004) [25]. Orphan drugs can also be conditionally approved; in this case, market exclusivity is granted only for the duration of 1 year. The price of (orphan) drugs is based on market-behavior, and therefore, the prices will reach to what the market is willing to pay for the drug [26]. Due to the relatively low amount of patients with an orphan disease, the price of orphan drugs is generally high [26–28]. In 2015, seven out of the top 10 best-selling drugs in the world had an orphan indication in the USA [29]. Considering that in certain chronic orphan diseases like cystic fibrosis, patients could use these expensive drugs for many decades. The total costs per patient could therefore become as high as several millions of euros. An example of such a high priced drug is eculizumab, a drug that treats paroxysmal nocturnal hemoglobinuria, which can cost up to $500,000 per patient per year [30].

### Drug repositioning in orphan disease

Recently, the public debate was excited by a price increase of a drug called mexiletine. This drug was originally developed 40 years ago for the treatment of arrhythmias. However, it was recently granted marketing authorization for 10 years after it was designated as orphan drug for non-dystrophic myotonia [31]. The price of mexiletine used to be €4.000 per patient per year but was increased to €80.000 per patient per year. The estimated costs for drug repositioning of mexiletine are clearly disproportional to the new price. This example shows that drug repositioning in orphan disease can result in disproportional and undesirable high prices. The primary incentives for drug development in orphan disease were not intended to serve drug repositioning and lead to overpricing of drugs. Thus, the legislation is not fitted for drug repositioning and should be adapted.

## Pharmaceutical compounding: an alternative?

Pharmaceutical compounding is the production of a medicinal product adapted to a specific need of a patient. Although authorized medicinal products are preferred, pharmacists can compound suitable medicinal products when the adequate commercial form or dosage is not available. However, the Directive 2001/83/EC states in Article 6 (1) that no medicinal product may be placed on the market of a member state unless a marketing authorization has been issued by the competent authorities of that member state or of the European Commission (EC). In Article 40 (1) of the Directive, it was established that manufacturing of the medicinal products is subject to the holding of license issued by the member states. Furthermore, the Directive states in Article 77 (1) that the wholesale distribution and storage are covered by an authorization granted by the member state according with this Directive. The exceptions to the above mentioned regulations can be found in Article (3) of Directive 2001/83/EC and shall not apply to:

1. Magistral formula: any medicinal product prepared in a pharmacy in accordance with a medical prescription for an individual patient [32].
2. Officinal formula: any medicinal product which is prepared in a pharmacy in accordance with the prescriptions of a pharmacopoeia and which is intended to be supplied directly to the patients served by the pharmacy in question [32].

These exceptions can only be applied when they are all in compliance with the requirements. In this case, then the marketing authorization, manufacturing, and wholesale licenses, as mentioned in the Directive, are not compulsory. The first requirement is that the medicinal product needs to be prepared in a pharmacy. Second, it needs to be prepared according to the clinician’s prescription. Lastly, the prescription must be made for an individual patient. In case the Directive does not require a marketing authorization for the medicinal product, EU member states are allowed to establish national regulations for pharmacy preparations.

Thus, the Directive allows exceptions such as pharmaceutical compounding of drugs for which an alternative medicinal product with a marketing authorization is available on the market [33].

### Pharmaceutical compounding; the case of chenodeoxycholic acid

More than 40 years ago, originally developed and registered for treatment of gallstones, chenodeoxycholic acid (CDCA) was found to be also effective treatment for cerebrotendinous xanthomatosis (CTX) [34, 35]. From 1976 until 2008, CDCA was available on the Dutch market for the treatment of gallstones under the brand name Chenofalk® which price was €0.28 per capsule. Chenofalk® has been used as off-label treatment for CTX since 1999, which costed at that time €308 per patient per treatment year. After CDCA was developed as Xenbilox® by Sigma-Tau, this company acquired the rights for Chenofalk® and later of Chenix®, a Belgian firm. By creating this monopoly position, Sigma-Tau gradually increased the price to around €29 per capsule. In 2015, CDCA was removed from the Dutch market for the treatment of gallstones [36, 37].

Subsequently, Sigma-Tau adopted a new name, Leadiant, and registered CDCA as an orphan drug for the treatment of CTX in 2017. The registration was based on two retrospective cohort studies [38]. Subsequently, the price of CDCA was increased to approximately €150.000 per patient per year on the Dutch market, a price increase of 500 times (see Table 2) [39, 40]. So, the company exploited the orphan drugs regulation (EU Regulation 141/2000) to raise the price and gain 10 years of market exclusivity [39]. This regulation for orphan drugs is meant to stimulate drug development for orphan diseases that would otherwise have no economic incentives to develop drugs for. However, CDCA was already being used for CTX for many years, and the estimated costs for preparing the registration file did not justify the steep raise in the price of CA. In April 2018, the Amsterdam UMC took the initiative to start magistral preparation of CDCA for its patient using raw materials imported from China. The costs were roughly €20.000 per patient per year, a fraction of the price of the drug sold by the pharmaceutical company [36]. Because of the high price [41] of the raw material, this magistral preparation is still 100 times higher than the original price of Chenofalk®.

**Table 2:** Price development of chenodeoxycholic acid from 2008 to 2017 [29]

Year	Brand name	Manufacturer	Price of 250 mg capsule	Price index
2008	Chenofalk	Dr. Falk	€0,28	1
2010	Xenbilox	Sigma-Tau	€8,67	31
2016	Xenbilox	Sigma-Tau	€36,53	130
2017	CDCA magistral	Amsterdam UMC	€22,83	81
2017	CDCA Leadiant	Leadiant	€140,00	500

### Drug repositioning in response to COVID-19

The spread of the novel coronavirus, SARS-COV 2, has caused a worldwide pandemic of the coronavirus disease 2019 (COVID-19). Since the start of the pandemic, drug repositioning research has been booming [42]. It has been found that the coronavirus has more than 60 druggable proteins that can be targeted by currently or previously approved drugs [43].

It is no doubt that drug repositioning will play an important role in the current COVID-19 pandemic [44]. The FDA has allowed the emergency use authorization for the unapproved product, remdesivir, for the treatment of severe hospitalized COVID-19 patients. Beigel et al. [45] reported superiority of remdesivir over placebo in shortening time to recovery in hospitalized patients. However, one Chinese study showed no clinical benefits of remdesivir [44]. Remdesivir was originally developed for hepatitis C and tested for the Ebola and Marburg viruses. It did not show to effective in any of these viral diseases [46, 47]. So, by definition, the case of remdesivir is not drug repositioning but shows that an earlier developed drug can be repurposed for a new indication.

More approved drugs have been identified to be potential candidates for drug repositioning for COVID-19 [48], and dozens of randomized clinical trials are currently conducted [49, 50]. A list of examples of these drugs can be found in Table 3. However, it is not clear yet whether these drugs with repositioning potential will be successful candidates for repositioning. The EMA has already announced that it will provide regulatory support to stimulate drug repositioning for COVID-19 [52]. It is necessary to provide such support to accelerate drug development for COVID-19; however, it should be a standard procedure to provide regulatory support to researchers that conduct drug repositioning for any other disease because otherwise unrealized potential of many future drugs is lost.

**Table 3:** List of drugs that are currently being clinically investigated as potential repositioned treatment for COVID-19 [51]

Drug	Mechanism of action	Original indication
Remdesivir	Viral RNA polymerase inhibitor	Hepatitis C
Favipiravir	Viral RNA polymerase inhibitor	Influenza
Hydroxychloroquine	MAPK inhibitor	Malaria
Lanadelumab	Inhibition of kallikrein	Hereditary angioedema
Ruxolitinib	JAK1/2 inhibitor	Myelofibrosis
Tocilizumab and sarilumab	IL-6 inhibitor	Rheumatoid arthritis
Anakira	IL-1ß-inhibitor	Rheumatoid arthritis
Lopinavir and ritonavir	Viral protease inhibitors	HIV
Oseltamivir	Neuraminidase inhibitor	Influenza
Imatinib	Abl kinase inhibitor	Leukemia
Cyclosporin A	Calcineurin inhibitor	Various (auto)-immune diseases
Nafamostat and camostat	TMPRSS2 inhibitor (viral membrane fusion inhibitor)	Anticoagulation
Enoxaparin and Rivaroxaban	Factor Xa inhibitor	Anticoagulation
Ravulizumab	Complement (C5) inhibitor	Paroxysmal nocturnal hemoglobinuria
Disulfiram	Inhibition of viral proteolysis	Chronic alcoholism
Ivermectin	Inhibition of viral replication	Onchocerciasis

## Discussion

Drug repositioning has the potential to offer benefits and new treatment options to patients. However, its potential has not been fully realized yet due to lack of incentives in the drug repositioning process. Therefore, patients might not have access to a beneficial repositioned drug or only through off-label prescriptions with its potential safety concerns and lack of availability for individual patients all over the world. In some cases of drug repositioning, pharmaceutical companies abused loopholes in the regulations to benefit from long-term regulatory exclusivity. This came hand-in-hand with extreme high pricing of repositioned drugs without the justifiable investments. The high pricing became subject of public debate, and for a well-balanced discussion, it is necessary that key players (e.g., policymakers, researchers) have insights into drug repositioning. The current lack of adequate national and European legislation causes therapeutic chances to be missed, not only in Europe, but also for the rest of the world.

To improve the drug repositioning process, cooperation between the pharmaceutical industry and clinical researchers is necessary for the efficient generation of data to determine the risks and benefits to register a new indication. For clinical researchers, it can be difficult to gain access to historical (non)-clinical data of the generic drugs; therefore, support of the original manufacturer is urged. In some instances, it would even require the researcher to repeat costly experiments or clinical studies again. Furthermore, clinicians should also collaborate together (e.g., by centers of expertise) to obtain adequate datasets for drug repositioning. This would ensure that these clinicians can operate more independently.

Clinical research groups may also have limited experience conducting trials to ensure that they meet regulatory requirements. This limited regulatory awareness and interest can hinder drug repositioning and can result in regulatory failures. Well-balanced regulatory support from national market authority agencies or the EMA is urgently needed for clinical research groups that intend to repurpose older drugs for new indications. These clinical research groups usually have extensive “real world” datasets that are valuable for regulatory authorities. The EMA and national regulatory authorities have shown to be dedicated to support (e.g., free rapid scientific advice and rapid compliance check) drug repositioning for COVID-19. The same level of support should be given to clinical research groups who intent drug repositioning. There needs to be a formal blueprint to bring this data into the regulatory process with an identified marketing authorization holder.

The COVID-19 pandemic has boosted worldwide drug repositioning initiatives. Rapid public and private funding became available for clinical groups to perform drug repositioning, and global data sharing initiatives were founded [53]. The COVID-19 pandemic has shown that it is possible to establish initiatives that stimulate drug repositioning and improve benefits for patients. The lessons learned from the COVID-19 pandemic should be implemented in a new standard blueprint for drug repositioning for all diseases.

## Conclusion

Drug repositioning has the potential to reregister older drugs for novel indications for the benefits of patients. It may provide new treatment opportunities for lower costs compared to de novo drug development. The current framework for drug repositioning allows “venture capital” companies to abuse loopholes in the legislation to gain long-term market authorization among with excessive high pricing. A new regulatory framework is needed to prevent abuse of the legislation and promote clinical investigator-driven drug repositioning. The COVID-19 pandemic has boosted funding and regulatory support for drug repositioning. The lessons learned from the COVID-19 pandemic should be implemented in a new standard blueprint for drug repositioning.

## Data Availability

No data was used to write this manuscript.
